# Supplementary material for: The neural correlates of shared and individual experience
Source: Commun Biol. 2026 Jan 21;9:95. doi: 10.1038/s42003-025-09355-3 (PMC12824322; doi:10.1038/s42003-025-09355-3)
Supplement: Supplementary file 2 — Supplementary Information [file 42003_2025_9355_MOESM2_ESM.pdf]

Supplementary Materials

The neural correlates of shared and individual experience.

Supplementary Material 1

Temporal autocorrelation bias in correlations between neural and behavioural time-by-time similarity matrix.

We had the object of detecting a correspondence between the subjective (i.e., suspense) and neural dynamics via the time-by-time similarity matrix (TTM; fig 1).

Given that the subjective and neural TTMs were obtained from different cohorts, and that we primarily wanted to investigate any correspondence between the two, we obtain a TTM for each sample (subjective and neural), by getting the average and the coefficient of variance across subjects. To estimate a significance value of such a correspondence we used a permutation analysis by which we shuffled the columns of the subjective TTM, corresponding to the timepoints of the story 10000 times (e.g., Kriegeskorte, 2015). This yielded a null distribution of correlation values arising out of TTM that have had their timepoints shuffled.

However, TTM have a characteristic degree of autocorrelation (see values along to diagonal fig. 1C and E). By autocorrelation, we here intend the heightened similarity between proximal timepoints, which are represented along the diagonal of the TTMs. It is natural that timepoints that are closer to each other will tendentially be more similar, both in terms of a stream of mental contents and in terms of neural states. Although this autocorrelational structure is relevant data to the present project (e.g., showing sharp transitions or a "lingering" in a state) this structure will increase correlation values between TTMs. However, of importance here is that a permuted TTM will not have this autocorrelational structure, therefore inflating p-values calculated via such a

permutation analysis. Therefore, to avoid a bias in significance probabilities we sought to remove autocorrelational values for the permutation analyses.

To establish how many autocorrelational values to remove for the permutation analysis we plotted the similarity between the subjective and neural TTM as a function of the amount of removed autocorrelation (proximal timepoint similarities) values (fig. S1A for average TTM and S1B for coefficient of variance TTMs). This was done by averaging across all networks for all participants in the awake condition. By getting the first derivative of the curves we have an illustration of how similarity decreased as a function of removed autocorrelation (fig. S1A and S1B), we found two points in which the similarity "stabilised", indicating decreased influence of autocorrelation (Fig. S1C for average and Fig S1D for coefficient of variance). The similarities stabilised by removing around 10 and 24 proximal time similarity values. These values were adopted for the permutation analyses. In the main text we present the correlation (Tau-A; τ_A) values between the full (no autocorrelation values removed) but the p-values of the permutation analyses of the TTM with 10 proximal time similarity values removed. Full results can be found in S2 (average TTM correspondence) and S3 (coefficient of variance TTM correspondence).

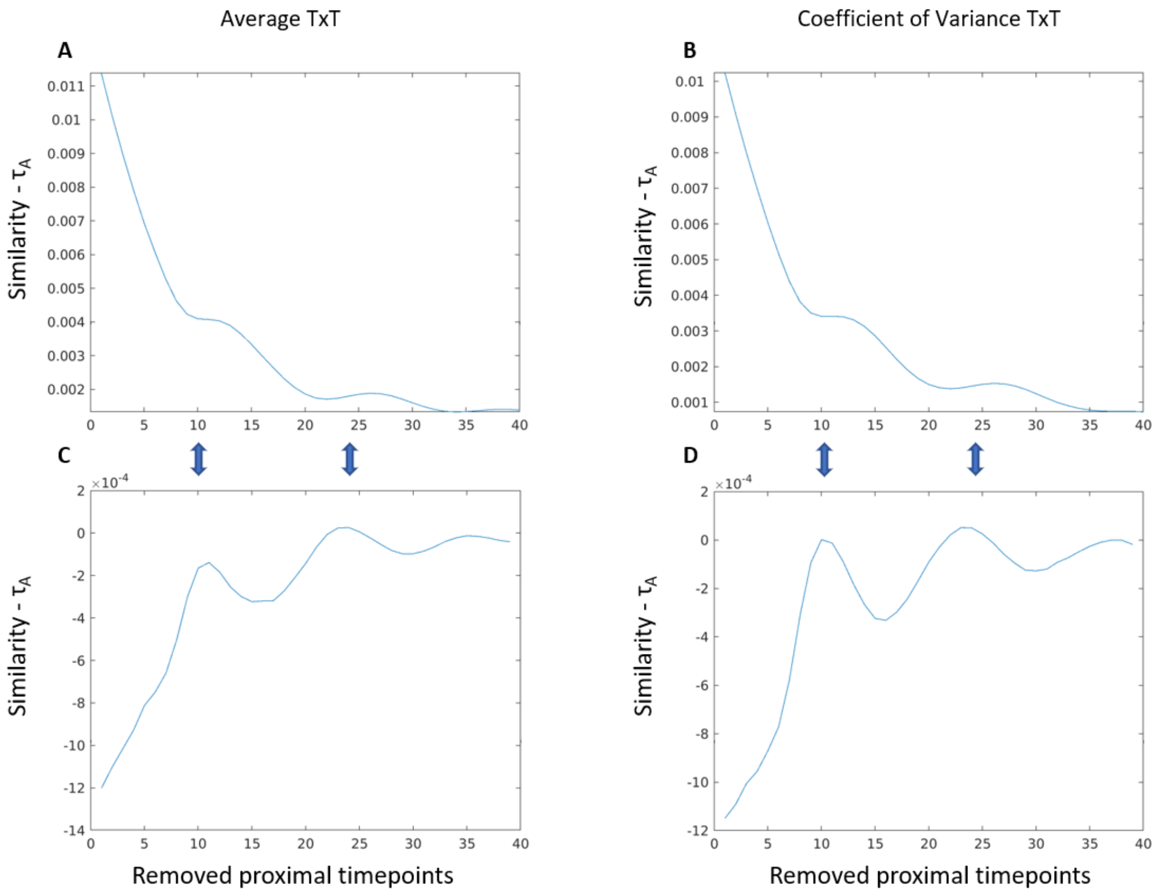

Figure S1. Similarity (Kendal's τ_A) as a function of removed autocorrelation.

A and B show the diminishing similarity (τ_A) as a function of removed proximal timepoint similarities (i.e., autocorrelation, values along the diagonal in TTM, e.g., see fig. 1C) for the similarity between subjective and neural average TTMs and Coefficient of Variance TTMs respectively. Figures C and D show the first derivative of A and B respectively revealing two points at which similarities stabilised despite increasing number of autocorrelation values removed. Blue bi-directional arrows indicate the points chosen (i.e., 10 and 24) for the permutation analyses in accordance with the stabilisation of similarities as a function of removed autocorrelation.

Supplementary Material 2

Similarity between neural and subjective average TTM for deconvolved and lagged BOLD data for all networks.

We sought to detect the correspondence between the average neural and subjective dynamics (i.e., TTM) by various means. As shown above we calculate p-values from the permutation analyses (n=10000) by removing 10 and 24 proximal timepoints. We account for hemodynamic delay via deconvolving the BOLD timeseries (see methods; Wu et al., 2021, 2013), the results for which are presented in the main text (Full results presented in table S1). To assess robustness of results we ran a confirmatory analysis where we introduce a delay in the BOLD signal (See Table S2) (Jääskeläinen et al., 2008; Khosla, Ngo, Jamison, Kuceyeski, & Sabuncu, 2021; Nishimoto et al., 2011), corresponding to the peak of the canonical hemodynamic response function (i.e., 6s) as implemented in SPM (https://www.fil.ion.ucl.ac.uk/spm/). Note, non-corrected p-values are presented here (corrected values presented in the main text).

Table S1. Deconvolved BOLD: Correlation (τ_A) and p-values for correspondence between neural and subjective average TTM.

Correlation (Tau-A) and permutation test values between the deconvolved neural TTM of each network (600 and 800 granularities) and the subjective suspense TTM averaged across participants. Presented are correlation values between the full subjective and neural TTMs as well as correlation and p values of TTM correspondence having removed 10 and 24 proximal timepoint similarities (see S1). These results are presented visually in figure S2.

Network Names	τ_A-full	τ_A-10	P-10	τ_A-24	P-24
DefaultA 600	0.32	0.21	<0.0001	0.15	<0.0001
DefaultA 800	0.33	0.22	<0.0001	0.16	<0.0001
DefaultB 600	0.22	0.10	0.0013	0.10	0.0178
DefaultB 800	0.19	0.05	0.0172	0.03	0.322
DefaultC 600	0.17	0.02	0.1857	0.03	0.1457
DefaultC 800	0.16	0.01	0.6003	0.01	0.5959
TempPar 600	0.07	-0.10	0.0225	-0.09	0.0086
TempPar 800	0.11	-0.04	0.3655	-0.06	0.1167
ContA 600	0.21	0.06	0.0041	0.03	0.2149
ContA 800	0.23	0.09	<0.0001	0.06	0.0233
ContB 600	0.17	0.02	0.442	-0.04	0.3239
ContB 800	0.23	0.09	<0.0001	0.03	0.0743
ContC 600	0.21	0.08	0.0357	0.03	0.1304
ContC 800	0.27	0.16	<0.0001	0.14	0.0002
LimbicA 600	0.18	0.04	0.2642	0.04	0.2536
LimbicA 800	0.16	0.01	0.6969	0.00	0.841
LimbicB 600	0.13	-0.04	0.566	-0.12	0.0038
LimbicB 800	0.18	0.02	0.2806	-0.05	0.1846
SalVentAttnA 600	0.27	0.15	<0.0001	0.12	0.0008
SalVentAttnA 800	0.29	0.17	<0.0001	0.15	0.0009
SalVentAttnB 600	0.16	0.00	0.8074	-0.01	0.6404
SalVentAttnB 800	0.18	0.02	0.0855	0.00	0.8317
DorsAttnA 600	0.17	0.02	0.144	0.00	0.8574
DorsAttnA 800	0.17	0.03	0.055	-0.02	0.4072
DorsAttnB 600	0.19	0.04	0.0554	-0.04	0.1405
DorsAttnB 800	0.21	0.07	0.003	-0.02	0.4702
SomMotA 600	0.24	0.10	0.0004	0.04	0.1557
SomMotA 800	0.26	0.13	0.0003	0.07	0.0653
Auditory 600	0.21	0.07	<0.0001	0.01	0.5789
Auditory 800	0.22	0.07	<0.0001	0.01	0.452
VisPeri 600	0.21	0.07	0.0445	0.02	0.3369
VisPeri 800	0.23	0.10	0.0056	0.06	0.0638
VisCent 600	0.21	0.09	0.0459	0.06	0.1183
VisCent 800	0.21	0.08	0.045	0.06	0.1138
SUB 54	0.18	0.06	0.0033	0.03	0.27

Table S2. Lagged BOLD: Correlation (τ_A) and p-values for correspondence between neural and subjective average TTM.

Correlation (Tau-A) and permutation test values between the lagged (6s) neural TTM of each network (600 and 800 granularities) and the subjective suspense TTM averaged across participants. Presented are correlation values (τ_A) between the full subjective and neural TTMs as well as correlation and p-values of TTM correspondence having removed 10 and 24 proximal timepoint similarities (see S1).

Network Names	τ_A-full	τ_A-10	P-10	τ_A-24	P-24
DefaultA 600	0.34	0.23	<0.0001	0.19	<0.0001
DefaultA 800	0.37	0.27	<0.0001	0.24	<0.0001
DefaultB 600	0.25	0.12	0.0004	0.12	0.0084
DefaultB 800	0.24	0.11	<0.0001	0.10	0.0042
DefaultC 600	0.15	-0.01	0.65	-0.04	0.2457
DefaultC 800	0.12	-0.05	0.0551	-0.07	0.011
TempPar 600	0.03	-0.15	0.005	-0.17	0.0001
TempPar 800	0.09	-0.07	0.4823	-0.10	0.0649
ContA 600	0.27	0.13	<0.0001	0.12	0.0025
ContA 800	0.27	0.14	<0.0001	0.13	0.0032
ContB 600	0.18	0.02	0.3211	-0.04	0.1427
ContB 800	0.22	0.08	<0.0001	0.02	0.3009
ContC 600	0.26	0.13	0.0095	0.10	0.0421
ContC 800	0.33	0.23	<0.0001	0.22	<0.0001
LimbicA 600	0.12	-0.05	0.0126	-0.06	0.0048
LimbicA 800	0.16	0.01	0.5991	-0.01	0.7094
LimbicB 600	0.10	-0.08	0.2697	-0.16	<0.0001
LimbicB 800	0.13	-0.05	0.7139	-0.12	0.0461
SalVentAttnA 600	0.22	0.08	0.0017	0.04	0.4022
SalVentAttnA 800	0.28	0.16	<0.0001	0.11	0.0824
SalVentAttnB 600	0.21	0.06	0.0699	0.06	0.2595
SalVentAttnB 800	0.23	0.08	0.0005	0.06	0.1141
DorsAttnA 600	0.28	0.15	<0.0001	0.16	<0.0001
DorsAttnA 800	0.25	0.11	0.0001	0.08	0.0177
DorsAttnB 600	0.22	0.08	0.0099	0.00	0.829
DorsAttnB 800	0.26	0.13	0.0005	0.08	0.0441
SomMotA 600	0.27	0.14	0.0001	0.08	0.0245
SomMotA 800	0.25	0.11	<0.0001	0.04	0.128
Auditory 600	0.20	0.06	0.0008	0.00	0.8884
Auditory 800	0.18	0.02	0.3616	-0.04	0.3477
VisPeri 600	0.25	0.11	0.0022	0.07	0.0568
VisPeri 800	0.23	0.09	0.0092	0.04	0.1336
VisCent 600	0.20	0.06	0.0967	0.01	0.5164
VisCent 800	0.25	0.12	0.0179	0.09	0.0437
SUB 54	0.16	0.02	0.1687	0.00	0.99

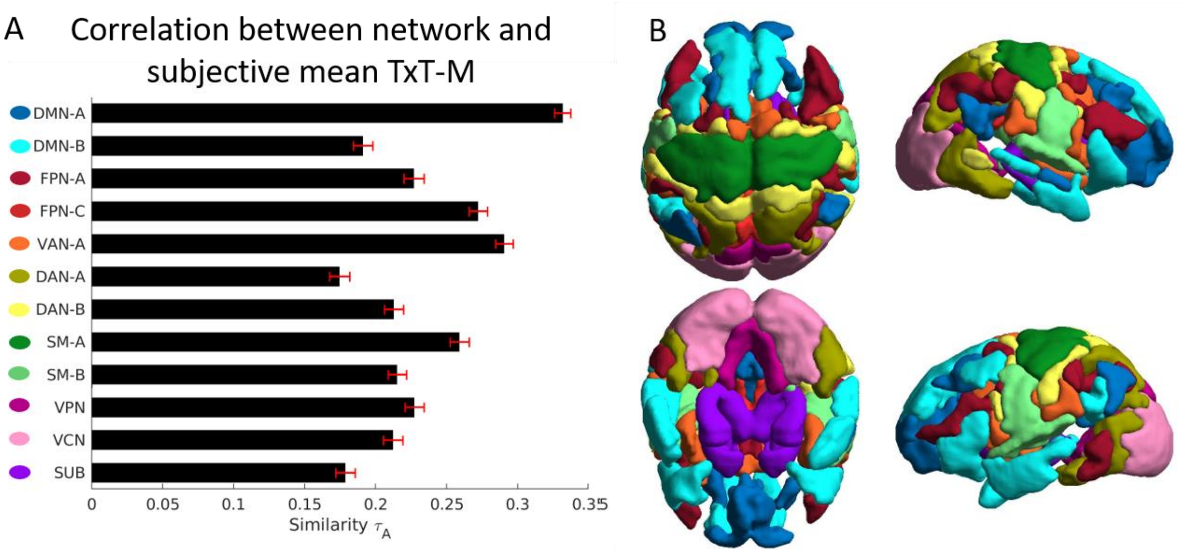

Figure S2. Correlation between network and subjective mean TTM and representation of networks. – Deconvolved.

The bar graph (A) represents similarities (τ_A) between the mean subjective and neural TTM for significant networks (see methods and S1). Error bars represent bootstrapped (n=1000) standard errors. (B) The significant networks are spatially represented in B. Colour legend for B can be found next to labels in A. DMN=Default Mode Network; FPN=Frontal Parietal Network; DAN=Dorsal Attention Network; VAN=Ventral Attention Network; SM=SomatoMotor Network (Auditory network in the main text); VCN=Visual Central Network; VPN= Visual Peripheral Network; SUB= Subcortex.

Of note is that although there is large correspondence between the deconvolved and lagged approaches, the convergence is not perfect. Namely the DAN-A and the Subcortex are significant in the Lagged and Deconvolved data respectively. However it is known that subcortical regions have abnormal haemodynamics compared to the cortex (Lewis, Setsompop, Rosen, & Polimeni, 2018; Miletić et al., 2020), potentially explaining the lack of consistency across different analyses. Hence the use of the blind deconvolution toolbox may have been beneficial for the analysis of the subcortex.

As for the DAN-A, it is beyond the scope of this study to investigate the differences between the lag (Jääskeläinen et al., 2008) and the deconvolution (Wu et al., 2013) method, and whether there is an interaction with the instantaneous phase synchrony method used in this study. Suffice it to say that in the deconvolution analysis the correlation values drop drastically (from 0.17

to 0.03) after autocorrelation is maintained whilst in the lagged analysis, they remain reasonably high after autocorrelation is removed (0.11, 0.15). This may be taken to suggest that in the lag analysis there is relevant information in the distal parts of the TTMs (i.e., which is not contained in the autocorrelation S1), whilst after deconvolution this is not the case.

Also interesting is that in the TemPar network, which showed a strikingly different behaviour between rest and story conditions (S5), showed negative correlations between the subjective and neural data once the autocorrelation was remove (Table S1 and S2).

Supplementary Material 3

Similarity between neural and subjective coefficient of variance TTM for deconvolved and lagged BOLD data for all networks.

We sought to investigate whether neural variability between subjects corresponded to subjective variability in temporal domain (as measured by TTM). We calculated the coefficient of variance across participants for the subjective and neural TTM and corresponded them using both the deconvolution and the lagged approach (See S2 and methods). The coefficient of variance is preferrable to standard deviation given the substantial difference in the nature of the subjective data (Likert scale from 1 to 9) compared to the neural data (Pearson correlation values from 1 to -1, between high dimensional connectivity matrices). Similarly, to the correspondence between average TTMs, p-values were calculated from a permutation analysis (n=10000) on TTMs that had the similarities between 10 and 24 proximal timepoints removed (see S1). Note, non-corrected p-values are presented here.

Table S3. Deconvolved BOLD: Correlation (τ_A) and p-values for correspondence between neural and subjective coefficient of variance TTM.

Correlation (Tau-A) and permutation test values between the coefficient of variance of the deconvolved neural TTM of each network (600 and 800 granularities) and the coefficient of variance of the subjective suspense TTM. Coefficient of variance was calculated across participants. Presented are correlation values (τ_A) between the full subjective and neural TTMs as well as correlation and p values of TTM correspondence having removed 10 and 24

proximal timepoint similarities (see S1). Significant results are also presented visually in figure S3.

Network Names	τ_A-full	τ_A-10	P-10	τ_A-24	P-24
DefaultA 600	0.33	0.23	<0.0001	0.2	<0.0001
DefaultA 800	0.35	0.27	<0.0001	0.24	<0.0001
DefaultB 600	0.21	0.1	0.0003	0.1	0.0099
DefaultB 800	0.16	0.03	0.1174	0.01	0.7607
DefaultC 600	0.14	0.01	0.7828	-0.01	0.5305
DefaultC 800	0.12	-0.02	0.6605	-0.03	0.4418
TempPar 600	0.08	-0.07	0.4163	-0.07	0.2521
TempPar 800	0.1	-0.04	0.7299	-0.05	0.4296
ContA 600	0.19	0.06	0.0059	0.04	0.1853
ContA 800	0.16	0.01	0.8964	-0.02	0.4306
ContB 600	0.17	0.04	0.0265	-0.02	0.3431
ContB 800	0.2	0.08	0.0011	0.02	0.3684
ContC 600	0.14	0.02	0.2818	-0.01	0.7026
ContC 800	0.19	0.07	0.01	0.08	0.008
LimbicA 600	0.13	0	0.9151	-0.02	0.1982
LimbicA 800	0.16	0.03	0.106	0.01	0.6325
LimbicB 600	0.15	0.01	0.7975	-0.07	0.1066
LimbicB 800	0.16	0.02	0.2368	-0.04	0.1491
SalVentAttnA 600	0.24	0.11	<0.0001	0.07	0.0228
SalVentAttnA 800	0.27	0.15	<0.0001	0.11	0.0038
SalVentAttnB 600	0.16	0.02	0.3981	-0.01	0.7158
SalVentAttnB 800	0.15	0	0.9206	-0.04	0.1908
DorsAttnA 600	0.16	0.02	0.3361	0	0.8665
DorsAttnA 800	0.16	0.02	0.2154	-0.01	0.3958
DorsAttnB 600	0.19	0.07	0.0164	0.01	0.6998
DorsAttnB 800	0.18	0.04	0.0421	-0.02	0.5124
SomMotA 600	0.18	0.04	0.0068	-0.02	0.3696
SomMotA 800	0.18	0.04	0.0417	0	0.9298
Auditory 600	0.21	0.09	<0.0001	0.06	0.0029
Auditory 800	0.24	0.12	<0.0001	0.1	0.0002
VisPeri 600	0.15	0.01	0.6151	-0.03	0.2053
VisPeri 800	0.2	0.07	0.0209	0.05	0.101
VisCent 600	0.22	0.1	0.0046	0.08	0.0383
VisCent 800	0.22	0.11	0.0056	0.1	0.0159
SUB 54	0.16	0.05	0.021	0.03	0.2936

Table S4. Lagged BOLD: Correlation (τ_A) and p-values for correspondence between neural and subjective coefficient of variance TTM.

Correlation (Tau-A) and permutation test values between the coefficient of variance of the lagged (6s) neural TTM of each network (600 and 800

granularities) and the coefficient of variance of the subjective suspense TTM. Coefficient of variance was calculated across participants. Presented are correlation values between the full subjective and neural TTMs as well as correlation and p-values of TTM correspondence having removed 10 and 24 proximal timepoint similarities (see S1).

Network Names	τ_A-full	τ_A-10	P-10	τ_A-24	P-24
DefaultA 600	0.31	0.22	<0.0001	0.21	<0.0001
DefaultA 800	0.33	0.24	<0.0001	0.24	<0.0001
DefaultB 600	0.2	0.07	0.1523	0.09	0.1468
DefaultB 800	0.2	0.07	0.0848	0.08	0.065
DefaultC 600	0.1	-0.06	0.0749	-0.09	0.0153
DefaultC 800	0.09	-0.07	0.0172	-0.09	0.007
TempPar 600	0	-0.18	<0.0001	-0.19	<0.0001
TempPar 800	0.07	-0.08	0.0761	-0.08	0.0844
ContA 600	0.25	0.12	0.0006	0.1	0.0395
ContA 800	0.22	0.08	0.0021	0.09	0.0102
ContB 600	0.21	0.08	0.0099	0.03	0.6332
ContB 800	0.23	0.11	0.0008	0.07	0.1631
ContC 600	0.18	0.05	0.2453	0.04	0.2209
ContC 800	0.26	0.15	0.0006	0.17	0.0007
LimbicA 600	0.11	-0.04	0.0687	-0.05	0.0224
LimbicA 800	0.19	0.06	0.0034	0.07	0.0041
LimbicB 600	0.14	-0.02	0.7682	-0.08	0.1003
LimbicB 800	0.14	-0.01	0.9523	-0.07	0.3489
SalVentAttnA 600	0.23	0.1	<0.0001	0.08	0.0057
SalVentAttnA 800	0.27	0.15	<0.0001	0.13	0.0005
SalVentAttnB 600	0.18	0.04	0.1485	0.05	0.3263
SalVentAttnB 800	0.19	0.04	0.0735	0.03	0.552
DorsAttnA 600	0.21	0.08	0.0036	0.07	0.0568
DorsAttnA 800	0.2	0.06	0.0306	0.04	0.3852
DorsAttnB 600	0.24	0.12	0.0014	0.08	0.1129
DorsAttnB 800	0.22	0.09	0.0142	0.05	0.1875
SomMotA 600	0.23	0.09	<0.0001	0.04	0.0889
SomMotA 800	0.19	0.05	0.0039	0.01	0.4947
Auditory 600	0.19	0.05	0.0003	0.02	0.247
Auditory 800	0.19	0.05	0.0002	0.01	0.3954
VisPeri 600	0.19	0.07	0.1551	0.04	0.5825
VisPeri 800	0.2	0.07	0.023	0.04	0.2727
VisCent 600	0.17	0.04	0.1585	0	0.8383
VisCent 800	0.26	0.15	0.0001	0.13	0.0027
SUB 54	0.14	0.02	0.2506	0.01	0.5783

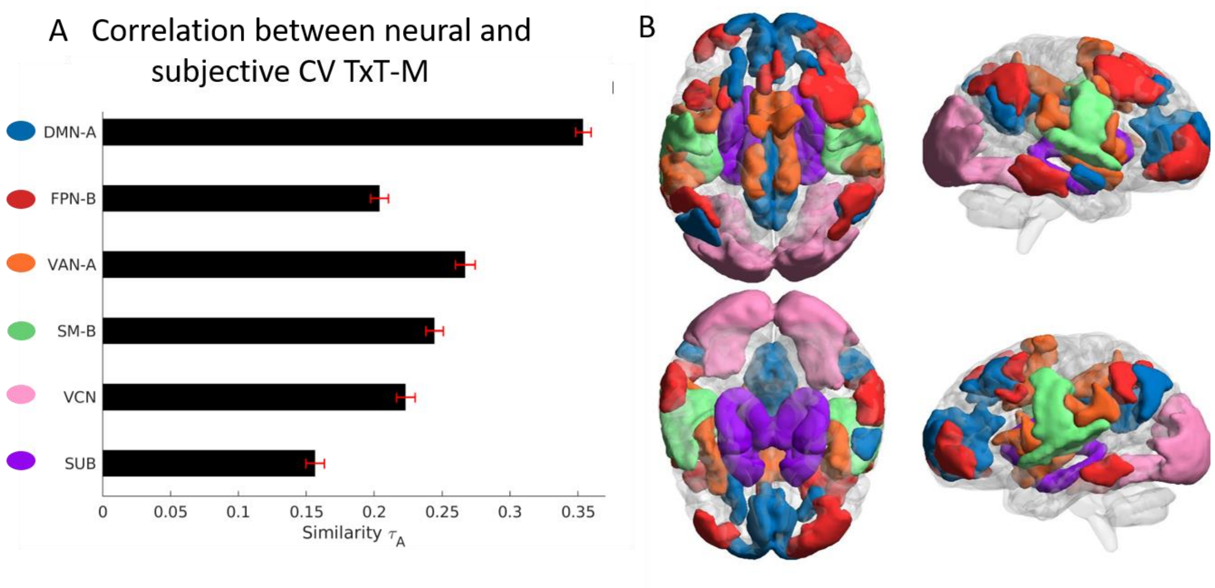

Figure S3. Correlation between network and subjective coefficient of variance TTM across individuals and representation of networks – Deconvolved.

The bar graph (A) represents similarity between the coefficient of variance neural and subjective TTM for significant networks. Error bars signify bootstrapped (n=1000) standard errors. The spatial localisations of these networks are presented (B). DMN=Default Mode Network; FPN=Frontal Parietal Network; VAN=Ventral Attention Network; SM=SomatoMotor Network (Auditory network in the main text); VCN=Visual Central Network; VPN= Visual Peripheral Network; SUB= Subcortex.

Once again, there was a good convergence between results. Particularly in the DMN A, SMN A and B, FPN-B, DAN-B.

However, this was not the case for the DMN C, the higher granularity FPN-A, the lower granularity VCN and similarly to the correspondence between average TTMs (S1) the subcortex and the DAN-A. Interestingly, it seemed that the lagged methods yielded more significant results in the correspondence between the variation across participants in neural and subjective TTMs.

Also, similarly to above the TemPar showed negative correlations between neural and subjective coefficient of variance TTMs once autocorrelation was removed. This, together with the average TTM correspondence analysis (S2) suggests that when the autocorrelation is removed, TemPar varies most when ratings of suspense are stable. This perhaps reflects a higher order, on-going processing that occurs in spite of stable feelings of suspense.

Supplementary Material 4

Planned contrasts of inter-subject similarities between awake and deep conditions during story listening for each network.

We established that there was a significant interaction of inter-subject similarity between the networks (n=18) and the level of anaesthesia (n=3). Therefore, we sought to explore which networks showed higher similarity in consciousness and higher dissimilarity in consciousness (compared to deep anaesthesia), taking this as evidence that these networks would be involved in general and special experiences respectively. We thus ran a Wilcoxon signed rank test on the inter-subject similarities (between each of the 16 participants, thus n=120) of the awake and deep conditions. Here we present the ensuing results for each of the networks across the two granularities.

Table S5. Wilcoxon signed rank z and p-values of difference between inter-subject similarities in awake and deep anaesthesia conditions.

Network Names	Z-value	P-value
DefaultA 600	-7.60	<0.0001
DefaultA 800	-6.84	<0.0001
DefaultB 600	-7.14	<0.0001
DefaultB 800	-8.49	<0.0001
DefaultC 600	-5.69	<0.0001
DefaultC 800	-3.79	0.0001
TempPar 600	-9.08	<0.0001
TempPar 800	-8.93	<0.0001
ContA 600	2.84	0.0045
ContA 800	1.87	0.0611
ContB 600	1.10	0.2714
ContB 800	-4.36	<0.0001
ContC 600	-9.05	<0.0001
ContC 800	-9.10	<0.0001
LimbicA 600	7.83	<0.0001
LimbicA 800	8.54	<0.0001
LimbicB 600	1.53	0.1249
LimbicB 800	3.39	0.0007
SalVentAttnA 600	-3.68	0.0002
SalVentAttnA 800	-3.31	0.0009
SalVentAttnB 600	-4.86	<0.0001
SalVentAttnB 800	-5.21	<0.0001
DorsAttnA 600	1.45	0.1461
DorsAttnA 800	3.14	0.0017
DorsAttnB 600	-0.05	0.9624
DorsAttnB 800	-0.05	0.9561
SomMotA 600	2.10	0.0362

SomMotA 800	2.80	0.0051
Auditory 600	5.09	<0.0001
Auditory 800	6.62	<0.0001
VisPeri 600	8.27	<0.0001
VisPeri 800	8.16	<0.0001
VisCent 600	-7.51	<0.0001
VisCent 800	-6.40	<0.0001
SUB 54	8.99	<0.0001

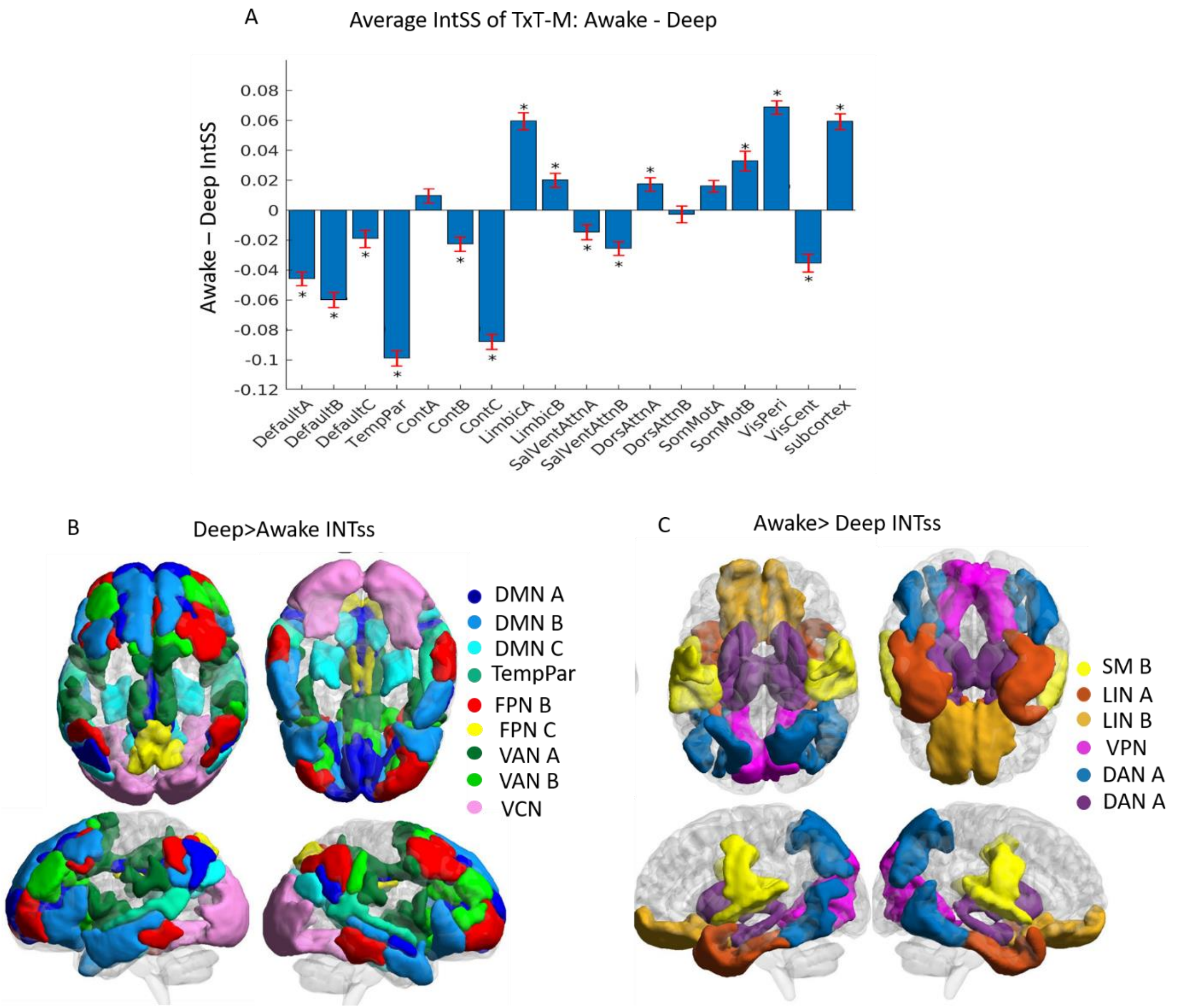

Figure S4. Inter-subject similarity differences between awake and deep anaesthesia.

(A) Awake – Deep average inter-subject similarity (IntSS) of TTMs for each network. Asterisks mark FWE corrected significance. Error bars signify bootstrapped (n=1000) standard error of mean difference. Maps showing which systems significantly display more dissimilarity during consciousness (Deep>Awake; B) and more inter-subject similarity (IntSS) during consciousness (Awake>Deep; C). Asterisks indicate FEW corrected significances. Error bars indicate bootstrapped (n=1000) standard error of mean difference.

Supplementary Material 5

3x2 ANOVA for each network. Inter-subject similarities in awake moderate and deep conditions during rest and story listening.

We wanted to ensure that the proposed "shared and individual" neural correlates of experiences are actually about the contents arising from listening to the story, rather than just a general effect of anaesthesia regardless of story condition. Therefore, we investigated if the inter-subject similarities in the different states of consciousness (awake, moderate anaesthesia, and deep anaesthesia) showed a different pattern of effects between resting state and story listening conditions. We thus built a 3x2 ANOVA (control, moderate and deep anaesthesia, for both rest and story listening) for each network. We particularly focused on the interaction effect in the main text, but here we present results for the two factors (consciousness state and rest vs story task). Of note is that the inter-subject similarities tended to be higher in the resting state condition which is counter intuitive. Upon further investigation, we found that the autocorrelation in the resting condition is typically much higher than the story condition. Perhaps this suggests that the story condition obliges the brain to track the stimuli dynamics more tightly, thus inducing quicker changes over proximal timepoints. This is an interesting finding in its own right, but beyond the scope of the present work.

Table S6. Values for 3x2 ANOVA for each network. F and P-values presented for interaction between consciousness condition and task vs rest, for task vs rest, and for consciousness condition (awake, moderate, deep anaesthesia).

Network Names	F-value Interaction	P-value Interaction	F-value Task vs Rest	P-value Task vs Rest	F-value consciousness condition	F-value consciousness condition
DefaultA 600	5.44	0.0045	6.23	0.0128	79.19	<0.0001
DefaultA 800	0.15	0.8603	1.08	0.2989	116.7	<0.0001
DefaultB 600	31.33	<0.0001	115.39	<0.0001	24.94	<0.0001
DefaultB 800	50.11	<0.0001	91.51	<0.0001	62.75	<0.0001
DefaultC 600	4.96	0.0073	11.38	0.0008	31.43	<0.0001
DefaultC 800	12.58	<0.0001	2.2	0.1387	28.53	<0.0001
TempPar 600	137.33	<0.0001	294.94	<0.0001	22.08	<0.0001
TempPar 800	124.05	<0.0001	365.52	<0.0001	11.07	<0.0001
ContA 600	21.7	<0.0001	19.57	<0.0001	9.43	0.0001
ContA 800	4.06	0.0177	13.23	0.0003	3.5	0.0306
ContB 600	24.31	<0.0001	27.94	<0.0001	14.81	<0.0001

ContB 800	3.24	0.0397	6.31	0.0122	32.44	<0.0001
ContC 600	31.74	<0.0001	82.87	<0.0001	68.85	<0.0001
ContC 800	46.29	<0.0001	93.16	<0.0001	98.87	<0.0001
LimbicA 600	12.42	<0.0001	0.64	0.4256	136.79	<0.0001
LimbicA 800	35.93	<0.0001	1.25	0.2643	170.74	<0.0001
LimbicB 600	3.51	0.0304	60.55	<0.0001	14.58	<0.0001
LimbicB 800	4.98	0.0071	39.25	<0.0001	31.09	<0.0001
SalVentAttnA 600	1.46	0.2322	1.11	0.2931	8.91	0.0002
SalVentAttnA 800	6.7	0.0013	5.56	0.0186	14.6	<0.0001
SalVentAttnB 600	17.85	<0.0001	116.68	<0.0001	3.9	0.0206
SalVentAttnB 800	9.24	0.0001	73.92	<0.0001	7.39	0.0007
DorsAttnA 600	6.77	0.0012	22.3	<0.0001	14.65	<0.0001
DorsAttnA 800	1.47	0.2302	30.83	<0.0001	20.43	<0.0001
DorsAttnB 600	2.34	0.0973	43.28	<0.0001	24.41	<0.0001
DorsAttnB 800	3.61	0.0276	23.11	<0.0001	35.04	<0.0001
SomMotA 600	6.16	0.0022	3.19	0.0743	30.88	<0.0001
SomMotA 800	13.56	<0.0001	0.04	0.8467	54.05	<0.0001
Auditory 600	6	0.0026	6.22	0.0128	20.47	<0.0001
Auditory 800	7.03	0.001	1.48	0.2237	27.63	<0.0001
VisPeri 600	15.3	<0.0001	4.55	0.0333	53.4	<0.0001
VisPeri 800	1.73	0.1781	5.35	0.021	78.33	<0.0001
VisCent 600	15.11	<0.0001	7.91	0.0051	53.55	<0.0001
VisCent 800	18.45	<0.0001	3.32	0.0689	28.74	<0.0001
SUB 54	1.72	0.1796	15.53	0.0001	109.22	<0.0001

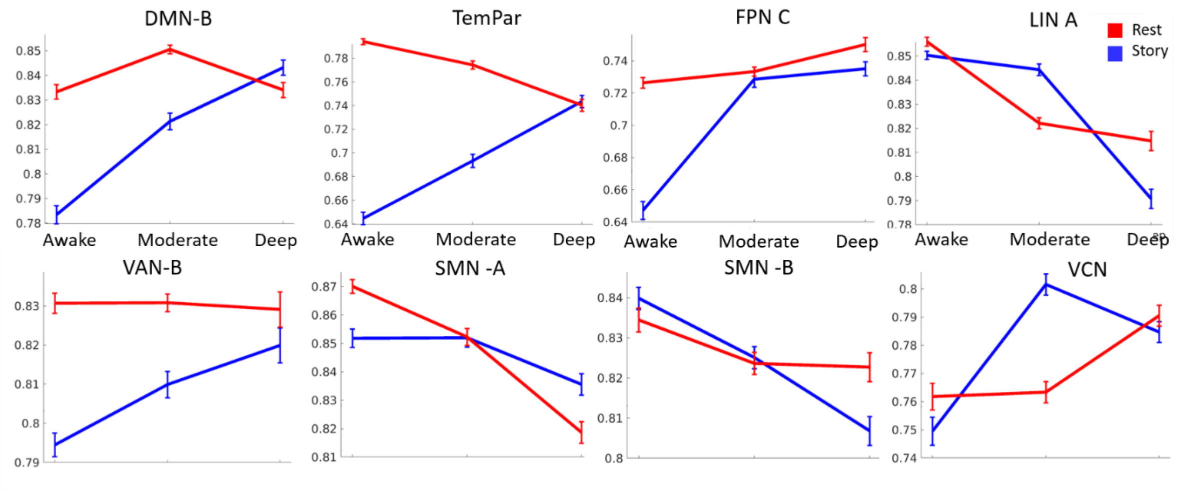

Figure S5. Rest-story interaction of inter-subject similarities as a function of level of anaesthesia

Significant interactions of inter-subject similarities from 3x2 ANOVA (Awake Moderate Deep [respectively presented from left to right], for story [Blue] and rest [Red] conditions).

Supplementary Material 6

Contrast of awake and deep TTM Shannon entropy during story listening for each network.

Finally, we investigated whether there were any differences between the awake and deep neural TTMs in terms of the complexity of their distribution. For this we used the built-in MATLAB function "entropy" (bins=256). Below are the results of the contrasts using Wilcoxon signed rank tests.

Table S7. Wilcoxon signed rank z and p-values of difference in Shannon entropy of TTMs between awake and deep anaesthesia conditions.

Network Names	Z-value	P-value
DefaultA 600	2.90	0.004
DefaultA 800	3.00	0.003
DefaultB 600	3.00	0.003
DefaultB 800	3.15	0.002
DefaultC 600	0.21	0.836
DefaultC 800	0.52	0.605
TempPar 600	3.21	0.001
TempPar 800	3.31	0.001
ContA 600	1.19	0.234
ContA 800	1.71	0.088
ContB 600	0.57	0.569
ContB 800	1.45	0.148
ContC 600	2.48	0.013
ContC 800	2.33	0.020
LimbicA 600	-1.81	0.070
LimbicA 800	-2.17	0.030
LimbicB 600	0.00	1.000
LimbicB 800	-0.10	0.918
SalVentAttnA 600	2.12	0.034
SalVentAttnA 800	2.07	0.039
SalVentAttnB 600	2.38	0.017
SalVentAttnB 800	2.17	0.030
DorsAttnA 600	-0.41	0.679
DorsAttnA 800	0.10	0.918
DorsAttnB 600	0.26	0.796
DorsAttnB 800	0.47	0.642
SomMotA 600	-1.76	0.079
SomMotA 800	-1.24	0.215
Auditory 600	-0.26	0.796
Auditory 800	-1.65	0.098
VisPeri 600	-1.40	0.163
VisPeri 800	-2.38	0.017
VisCent 600	-1.50	0.134
VisCent 800	-0.93	0.352
SUB 54	-3.05	0.002

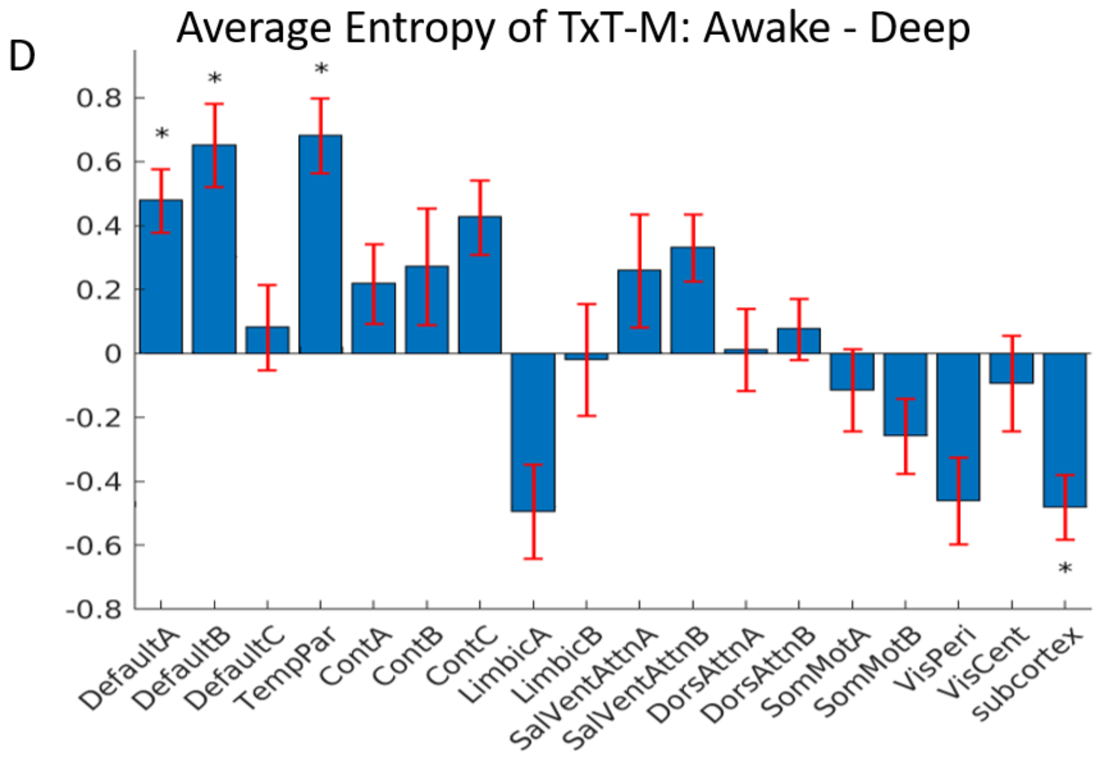

Figure S6. Shannon entropy differences between awake and deep anaesthesia.

Average Shannon entropy difference (Awake-Deep) of TTMs for each network. Error bars signify bootstrapped (n=1000) standard error of mean difference. Maps showing which systems significantly display more dissimilarity during consciousness (Deep>Awake; B) and more inter-subject similarity (IntSS) during consciousness (Awake>Deep; C). Asterisks indicate FEW corrected significances. Error bars indicate bootstrapped (n=1000) standard error of mean difference.

Note, for the correlation between the difference in intersubject similarity between consciousness and unconsciousness and the difference between complexity of the TTMs in consciousness and unconsciousness, we used all available networks (i.e., 18), as presented throughout the supplementary materials. The reason for this is that this would give a more reliable statistical estimate, instead of using the 4 networks that are reported in the main text.

Supplementary Material 7

Summary of results and supplementary discussion to the results not discussed in the main text.

We here review all results together to assess other possible neural correlates of general and special experiences during story listening. The are many results, and with several methodological contingencies (see methods) which we explored extensively (e.g., S1 S2 S3). Therefore, we have placed a summary of all the results in table S8. This shows analyses (columns) results for each network (Rows), and indicates with a tick (✓) If there was strong evidence, and a tilde (~) for moderate evidence for that analysis across analytical contingencies (see methods and supplementary materials for details).

In the supplementary discussion below, we will review each network that is proposed in the neural correlates of general and special experience in relation to previous research.

Table S8. Summary of results for each network across all analyses (see methods and supplementary materials) and inclusion into proposed neural correlates of general or special experience. The columns represent different analyses, presented above; whilst the rows networks (n=18). The final column represents whether the network was included into the neural correlates of general or special experience. A tick (✓) signifies strong convergence of evidence across analyses (See methods and supplementary materials) whilst a tilde (~) represents moderate evidence. Empty cells represent little or no evidence in specific analysis (corresponding to column). Networks and parcellations have been previously described (A. Schaefer et al., 2018; Yeo et al., 2011). In parentheses are the relevant supplementary materials for each type of analysis.

	**Subjective ∝ Neural Correspondence (average) (S2)**	**Subjective ∝ Neural correspondence Variation (S3)**	**Greater or lower IntSS in consciousness. (S4)**	**Story specific effects (Interaction with rest) (S5)**	**Possible neural correlates of general and special experience**
**DMN A**	✓	✓	✓		**SPECIAL**
**DMN B**	✓		✓	✓	**SPECIAL**
**DMN C**		~	✓		

**TemPar**			✓	✓	**SPECIAL**
**FPN A**	✓	~			
**FPN B**		✓			
**FPN C**	✓		✓	✓	**SPECIAL**
**LIN A**			✓	✓	**GENERAL**
**LIN B**			✓		
**VAN A**	✓	✓	✓		**SPECIAL**
**VAN B**			✓	✓	**SPECIAL**
**DAN A**	~	~	~		**GENERAL**
**DAN B**	✓	✓			
**SM A**	✓	✓		✓	**GENERAL**
**AUD**	✓	✓	✓	✓	**GENERAL**
**VPN**	✓		✓		**GENERAL**
**VCN**		~	✓	✓	**SPECIAL**
**SUB**	~	~	✓		**GENERAL**

Supplementary discussion.

In this supplementary section, we review the results of the networks not discussed in the main text in relation to previous results.

Our main objective was to propose preliminary neural correlates of general and special experience. Given our exploratory results, evidencing involvement in the task and higher inter-subject dissimilarity in consciousness, we suggest that, beyond the DMN, the ventral attention network (VAN) dynamics are involved in individual specific experience (For reference see figures S2, S3, S4 and S5; or table S8). Furthermore, it seems that a precuneal component of the frontal parietal control network (FPN-C) and the early visual network also display individual-specific dynamics. Conversely, given relevance to the task and higher

similarity in consciousness, the dynamics of the subcortex, the visual peripheral network, the somato-motor network and Limbic A all seem to support relatively more generalisable streams of consciousness across the different individual experiences. We shall now briefly discuss these networks in relation to previous literature.

Adjacent to the posterior DMN-A regions, the FPN-C also includes a dorsal precuneal and a ventral posterior cingulate region (sometimes included in the DMN (Cavanna, 2007; Lyu, Pappas, Menon, & Stamatakis, 2021). These precuneal and cingulate regions are thought to compose an integratory hub supporting the most complex cognition (Cavanna, 2007; Leech, Braga, & Sharp, 2012; Lyu et al., 2021; Margulies et al., 2016; van den Heuvel & Sporns, 2011). Such posterior regions have also been found to track the narrative of stories at their most abstract levels, so much so that they are generalisable across modalities (Baldassano, Chen, Zadbood, Pillow, & Norman, 2017; Chen et al., 2017). This network was found to track the average of the subjective TTM and to display a differential effect between rest and story listening as a function of (un)consciousness (S2, S4 and Table S8).

The temporal parietal network was originally included in the DMN (in 7 network definition as opposed to the 17 network definition) and comprises superior temporal and parietal regions (angular gyrus specifically). These, similarly to the DMN-B discussed in the main text, are regions commonly associated with language related processing. In fact, when compared to neurosynth meta-analitic maps, this network had a substantial overlap with maps associated with the term “language” (57.6 %) and “semantic” (36%). Thus, broadly speaking, interpretations are equivalent to those proposed for the DMN-B in the main text. However, see text at the end of supplementary materials 2 and 3 for additional interesting findings regarding the temporal parietal network.

Conversely to the DMN -B and the TemPar, the limbic A network is also comprised of temporal regions (inferior and anterior pole), but is seemingly involved in common aspects of experience. Also a higher order network (Margulies et al., 2016) these regions are known to be involved in object recognition, semantic processing and social cognition (Conway, 2018; Lambon Ralph, Jefferies, Patterson, & Rogers, 2016; Pehrs et al., 2015), which are known to be affected by propofol sedation (Adapa, Davis, Stamatakis, Absalom, & Menon, 2014). Thus, whilst the inferior temporal ventral stream, known to be involved in object recognition (e.g., Conway, 2018; Pehrs et al., 2015) may underlie common experiences, the more middle temporal and angular gyri (DMN-B and TemPar) may underlie more individual-specific semantically and mnemonically driven experiences (Chen et al., 2017; Friederici, 2011; Hagoort, 2019; Smallwood et al., 2016).

Of less easy interpretation is the involvement in the ventral attention network (VAN; also called salience in Schaefer et al., 2018; Yeo et al., 2011). Interpreted as being involved in stimulus-driven attention (Vossel, Geng, & Fink, 2014), the VAN-A’s dynamics has shown correlations to suspense dynamics (fig. S2), but also the tracking of individual differences, second only to the DMN-A (fig. S3). The VAN B instead showed to behave differently in the story versus rest conditions, showing virtually no effect in IntSS resting-state effects across levels of anaesthesia, but a substantial scaling during story listening (fig. S4). Although “stimulus-driven attention” would indicate common experience (the stimulus being the

commonality), what counts as a “salient” stimulus shows very large and consistent individual differences (De Haas, Iakovidis, Schwarzkopf, & Gegenfurtner, 2019). Furthermore, this network’s dynamics may reportedly also be driven by “internal” memory-driven stimuli, (Corbetta, Patel, & Shulman, 2008; Seeley, 2019; Vossel et al., 2014), which will be specific to the individual’s development. In fact pathological (Seeley, 2019) and personality differences (Tian, Wang, Xu, Li, & Ma, 2018) have been found in relation to this network, indicating that it may underlie a substantial portion of individual differences.

Thus, beyond the classic DAN-DMN antithesis discussed in the main text (Huang, Zhang, Wu, Mashour, & Hudetz, 2020), another dynamic balance may underlie individual and common experiences: The ventral and dorsal attention networks respectively. These networks, described as being circuit breakers for one another (Corbetta et al., 2008) engage in a dynamic and flexible interplay (Vossel et al., 2014), that would tendentially support special and common attention-driven experiences.

Conversely to the auditory network reported in the main text, the correlation with suspense dynamics of story with visual and somato-motor dynamics perhaps may be counterintuitive. However, it has been repeatedly shown that information in one sensory modality may instigate processing in other sensory modalities (Adolphs, Damasio, Tranel, Cooper, & Damasio, 2000; Akama, Murphy, Na, Shimizu, & Poesio, 2012; De Borst & De Gelder, 2017; Pulvermuller, 2005; Sanchez, Hartmann, Fusca, Demarchi, & Weisz, 2020; M. Schaefer, Rotte, Heinze, & Denke, 2015; Vetter, Smith, & Muckli, 2014). The complete irrelevance of the visual and motor system in representing auditory information is unlikely. For example, visual peripheral network (VPN) dynamics are known to be primed by attention/stimuli-salience in a behaviourally relevant way (Cate et al., 2009) as well as it’s dynamics potentially being relevant to content imagery (Vetter et al., 2014), which may be of particular interest to the auditory story condition.

The involvement of the SMN-A may also be controversial. However its emotional processing in naturalistic social settings (Adolphs et al., 2000; Kropf, Syan, Minuzzi, & Frey, 2019; M. Schaefer et al., 2015) and in auditory imagery (De Borst & De Gelder, 2017) is well documented, suggesting a role in embodied representations. In fact, several bodily actions are inferable from the auditory story presented to participants. The Visual Central Network, is a surprising finding for the neural correlates of special experience (see table S8). One potential explanation is differences between individual’s use of the early visual cortex during an auditory story (Reeder, 2017; Vetter et al., 2014). Differences may lie in imagery vividness, content of visualisation and visual/behavioural priming strategies (e.g., Cate et al., 2009; Reeder, 2017; Zeman, Dewar, & Della Sala, 2015). Nonetheless, its involvement in the task is evidenced by the interaction analysis (fig. S5) and its correlation with the average and the variance of the subjective dynamics (fig. S2 and S3).

Finally, The subcortex is the oldest and physiologically most fundamental network to be included in this study (Panksepp, 2011). It is well suited to underlie common experiences in that it supports survival related processes. In fact, we found that it’s dynamics may track that of subjective feelings of suspense and also that its variations across individuals may potentially track individual differences in subjective reporting (Although note, only with the

deconvolution method rather than the lagged method, see S2). Although our data (fig. S5) suggests that the subcortical dynamics are more similar in consciousness compared to unconsciousness, we cannot discount experience-driven individual differences in subcortical processing (e.g., Péron, Frühholz, Ceravolo, & Grandjean, 2015; Sylvester et al., 2020; Telzer et al., 2013). An interesting finding, is that the subcortex is the only network that consistently displayed increased complexity in the unconscious condition (fig. S6), perhaps indicating a disorganisation in dynamics; the organisation of which might be relevant to consciousness.

Finally, although our analyses permitted a relative categorisation of a network as underlying general or special experiences, as mentioned in the main text, this rough division is likely to be exposed as unimaginably more intricate as new techniques and paradigms permitting a concurrent analysis at multiple dimensions (experiential, spatial and temporal) are developed. This may partly explain why the FPN-A, despite being involved in the task and showing a trend in heightened similarity in consciousness, did not display sufficient evidence to be included in general neural correlates of experience (Naci et al., 2018; Naci, Sinai, & Owen, 2017).

Another interesting finding in relation to this is that the network dynamics that display increased individual differences in consciousness tend to contain more information (as measured by Shannon entropy), whilst the dynamics that are more similar between individuals in consciousness are relatively more structured. Increased information of intrinsic dynamics would naturally result in less inter-subject similarity. However, this finding may also elucidate the dynamic equilibrium between more personal-subjective and more communicable-objective processes that the brain has to constantly negotiate (Carhart-Harris & Friston, 2010; Friston, 2010). Presumably, certain pathologies may be partially explained by a deviation of such an equilibrium (Carhart-Harris & Friston, 2019; Froggatt, 2005; Fromm, 1998). In fact, in line with typical notions of consciousness (Carhart-Harris et al., 2014) the control awake condition tended to show more entropy over all networks (fig. S6). Only four networks showed a family-wise error corrected effect across granularity. The DMN-A, DMN-B and Temporal Parietal networks showed higher entropy during the consciousness condition, whilst the subcortex showed the opposite effect (see S6 for full results).

Another apparently contradictory result in relation to previous work, is the higher subcortical dynamics complexity (measured via Shannon Entropy) in unconsciousness. Previous results (Coppola et al., 2022) show heightened complexity of subcortical dynamics by measuring the temporal information of various graph theory properties and intrinsic dynamics in rest. However, while these papers measured complexity of temporal sequences, here we applied Shannon entropy looking at the complexity of the distribution of intrinsic similarity (which is shown to diminish in consciousness in proximal transitions, Coppola et al., in press). Thus, here we discard temporally-specific information of moment-to-moment changes and focus on the absolute variation of intrinsic similarity values. This suggests that despite having a higher complexity in temporal sequences in consciousness (resting state), the subcortex may display a more organised structure in the long-term temporal distribution during such a story (S9; e.g., visiting similar “states”, or travelling similar distances overtime), which may translate

to a particularly predictive desynchronisation in unconsciousness (Tsurugizawa & Yoshimaru, 2021; Coppola et al., 2022).
