# Supplementary material for: The neural correlates of shared and individual experience
Source: Commun Biol. 2026 Jan 21;9:95. doi: 10.1038/s42003-025-09355-3 (PMC12824322; doi:10.1038/s42003-025-09355-3)
Supplement: Supplementary file 3 — Reporting Summary [file 42003_2025_9355_MOESM3_ESM.pdf]

Corresponding author(s): Peter Coppola
Last updated by author(s): 28/11/25

Reporting Summary

Nature Portfolio wishes to improve the reproducibility of the work that we publish. This form provides structure for consistency and transparency in reporting. For further information on Nature Portfolio policies, see our Editorial Policies and the Editorial Policy Checklist.

Please do not complete any field with "not applicable" or n/a. Refer to the help text for what text to use if an item is not relevant to your study.
For final submission: please carefully check your responses for accuracy; you will not be able to make changes later.

Statistics

For all statistical analyses, confirm that the following items are present in the figure legend, table legend, main text, or Methods section.

n/a	Confirmed	
☐	☑	The exact sample size (n) for each experimental group/condition, given as a discrete number and unit of measurement
☐	☑	A statement on whether measurements were taken from distinct samples or whether the same sample was measured repeatedly
☐	☑	The statistical test(s) used AND whether they are one- or two-sided<br>*Only common tests should be described solely by name; describe more complex techniques in the Methods section.*
☐	☑	A description of all covariates tested
☐	☑	A description of any assumptions or corrections, such as tests of normality and adjustment for multiple comparisons
☐	☑	A full description of the statistical parameters including central tendency (e.g. means) or other basic estimates (e.g. regression coefficient) AND variation (e.g. standard deviation) or associated estimates of uncertainty (e.g. confidence intervals)
☐	☑	For null hypothesis testing, the test statistic (e.g. F, t, r) with confidence intervals, effect sizes, degrees of freedom and P value noted<br>*Give P values as exact values whenever suitable.*
☒	☐	For Bayesian analysis, information on the choice of priors and Markov chain Monte Carlo settings
☒	☐	For hierarchical and complex designs, identification of the appropriate level for tests and full reporting of outcomes
☐	☑	Estimates of effect sizes (e.g. Cohen's d, Pearson's r), indicating how they were calculated

Our web collection on statistics for biologists contains articles on many of the points above.

Software and code

Policy information about availability of computer code

Data collection	Typical code for MRI data collections was used. See Naci et al., 2018 and the present paper for further detail.
Data analysis	Relevant code can be found here https://github.com/Peter6789/Neural-correlates-of-individual-and-shared-experience. Further information in the paper.

For manuscripts utilizing custom algorithms or software that are central to the research but not yet described in published literature, software must be made available to editors and reviewers. We strongly encourage code deposition in a community repository (e.g. GitHub). See the Nature Portfolio guidelines for submitting code & software for further information.

Data

Policy information about availability of data

All manuscripts must include a data availability statement. This statement should provide the following information, where applicable:
- Accession codes, unique identifiers, or web links for publicly available datasets
- A description of any restrictions on data availability
- For clinical datasets or third party data, please ensure that the statement adheres to our policy

Research involving human participants, their data, or biological material

Policy information about studies with human participants or human data. See also policy information about sex, gender (identity/presentation), and sexual orientation and race, ethnicity and racism.

Reporting on sex and gender	Sex and gender were not a variable of interest. Further information can be found in Deng et al., 2023 for behavioural data and Naci et al., 2018 for fMRI data and in the manuscript.
Reporting on race, ethnicity, or other socially relevant groupings	This was not a variable of interest. Further information can be found in Deng et al., 2023 for behavioural data and Naci et al., 2018 for fMRI data and in the manuscript.
Population characteristics	Repeated measures design meant no covariates were inserted. Further information can be found in Deng et al., 2023 for behavioural data, Naci et al., 2018 for fMRI data and in the manuscript. .
Recruitment	All experiments were performed in accordance with the relevant guidelines and regulations set out by the research ethics boards. All healthy volunteers were right-handed, native English speakers, and had no history of neurological disorders. Further information can be found in Deng et al., 2023 for behavioural data and Naci et al., 2018.
Ethics oversight	Ethical approval was obtained from the Health Sciences Research Ethics Board and Psychology Research Ethics Board of Western University.

Note that full information on the approval of the study protocol must also be provided in the manuscript.

Field-specific reporting

Please select the one below that is the best fit for your research. If you are not sure, read the appropriate sections before making your selection.

☑ Life sciences ☐ Behavioural & social sciences ☐ Ecological, evolutionary & environmental sciences

For a reference copy of the document with all sections, see nature.com/documents/nr-reporting-summary-flat.pdf

Life sciences study design

All studies must disclose on these points even when the disclosure is negative.

Sample size	No power analysis was performed. Further information can be found in Deng et al., 2023 for behavioural data and Naci et al., 2018 for fMRI data and in the manuscript..
Data exclusions	Due to technical malfunction 3 participants were excluded.
Replication	Alternative analyses were performed. Namely using different parcellations with different granularities, and using alternative methodologies to correct for hemodynamics, as well as different cut offs for temporal autocorrelation (S1). Results had to be consistent and survive FDR.
Randomization	The fMRI experiment used a repeated measures design. The anaesthetic protocol meant that randomization was not possible. This is discussed in the manuscript.
Blinding	Blinding was not possible due to the anaesthetic procedure and safety concerns.

Behavioural & social sciences study design

All studies must disclose on these points even when the disclosure is negative.

Study description	NA
Research sample	NA
Sampling strategy	NA
Data collection	NA
Timing	NA
Data exclusions	NA
Non-participation	NA
Randomization	NA

Ecological, evolutionary & environmental sciences study design

All studies must disclose on these points even when the disclosure is negative.

Study description	NA
Research sample	NA
Sampling strategy	NA
Data collection	NA
Timing and spatial scale	NA
Data exclusions	NA
Reproducibility	NA
Randomization	NA
Blinding	NA

Did the study involve field work? ☐ Yes ☑ No

Field work, collection and transport

Field conditions	
Location	
Access & import/export	
Disturbance	

Reporting for specific materials, systems and methods

We require information from authors about some types of materials, experimental systems and methods used in many studies. Here, indicate whether each material, system or method listed is relevant to your study. If you are not sure if a list item applies to your research, read the appropriate section before selecting a response.

Materials & experimental systems

n/a	Involved in the study	
☐	☐	Antibodies
☐	☐	Eukaryotic cell lines
☐	☐	Palaeontology and archaeology
☐	☐	Animals and other organisms
☐	☐	Clinical data
☐	☐	Dual use research of concern
☐	☐	Plants

Methods

n/a	Involved in the study	
☐	☐	ChIP-seq
☐	☐	Flow cytometry
☐	☐	MRI-based neuroimaging

Antibodies

Antibodies used	NA
Validation	NA

Eukaryotic cell lines

Policy information about cell lines and Sex and Gender in Research

Cell line source(s)	NA
Authentication	NA
Mycoplasma contamination	NA
Commonly misidentified lines (See ICLAC register)	NA

Palaeontology and Archaeology

Specimen provenance	NA
Specimen deposition	NA
Dating methods	NA

☐ Tick this box to confirm that the raw and calibrated dates are available in the paper or in Supplementary Information.

Ethics oversight	NA

Note that full information on the approval of the study protocol must also be provided in the manuscript.

Animals and other research organisms

Policy information about studies involving animals; ARRIVE guidelines recommended for reporting animal research, and Sex and Gender in Research

Laboratory animals	NA
Wild animals	NA
Reporting on sex	NA
Field-collected samples	NA
Ethics oversight	NA

Note that full information on the approval of the study protocol must also be provided in the manuscript.

Clinical data

Policy information about clinical studies
All manuscripts should comply with the ICMJE guidelines for publication of clinical research and a completed CONSORT checklist must be included with all submissions.

Clinical trial registration	NA
Study protocol	NA
Data collection	NA
Outcomes	NA

Dual use research of concern

Policy information about dual use research of concern

Hazards

Could the accidental, deliberate or reckless misuse of agents or technologies generated in the work, or the application of information presented in the manuscript, pose a threat to:

No	Yes	
x	☐	Public health
x	☐	National security
x	☐	Crops and/or livestock
x	☐	Ecosystems
x	☐	Any other significant area

Experiments of concern

Does the work involve any of these experiments of concern:

No	Yes	
x	☐	Demonstrate how to render a vaccine ineffective
x	☐	Confer resistance to therapeutically useful antibiotics or antiviral agents
x	☐	Enhance the virulence of a pathogen or render a nonpathogen virulent
x	☐	Increase transmissibility of a pathogen
x	☐	Alter the host range of a pathogen
x	☐	Enable evasion of diagnostic/detection modalities
x	☐	Enable the weaponization of a biological agent or toxin
x	☐	Any other potentially harmful combination of experiments and agents

Plants

Seed stocks	NA
Novel plant genotypes	NA
Authentication	NA

ChIP-seq

Data deposition

☐ Confirm that both raw and final processed data have been deposited in a public database such as GEO.

☐ Confirm that you have deposited or provided access to graph files (e.g. BED files) for the called peaks.

Data access links<br>*May remain private before publication.*	
Files in database submission	
Genome browser session<br>(e.g. UCSC)	

Methodology

Replicates	
Sequencing depth	
Antibodies	
Peak calling parameters	
Data quality	

Software

Flow Cytometry

Plots

Confirm that:

- [ ] The axis labels state the marker and fluorochrome used (e.g. CD4-FITC).
- [ ] The axis scales are clearly visible. Include numbers along axes only for bottom left plot of group (a 'group' is an analysis of identical markers).
- [ ] All plots are contour plots with outliers or pseudocolor plots.
- [ ] A numerical value for number of cells or percentage (with statistics) is provided.

Methodology

Sample preparation

Instrument

Software

Cell population abundance

Gating strategy

- [ ] Tick this box to confirm that a figure exemplifying the gating strategy is provided in the Supplementary Information.

Magnetic resonance imaging

Experimental design

Design type: Block design (naturalistic stimuli) and resting state.

Design specifications: 3 levels of anesthesia. In each participants had a resting state condition and a condition in which a naturalistic auditory story was presented.

Behavioral performance measures: Response time for auditory stimuli under mild sedation. 3 participants were not responsive.

Imaging type(s): Functional and structural

Field strength: 3 tesla

Sequence & imaging parameters: TR was 2s, whilst TE was 30ms. Flip-angle was 75 degrees. There were 33 slices, with a 25% interslice gap. Resolution was 3mm, isotropic. Anatomical T1 images were also acquired 1mm isotropic voxels. Use of an MPRAGE sequence with 9 degrees FA, a matrix of 240x256, TE of 4.25ms.

Area of acquisition: Whole brain scan. Brain network definitions were used. Cerebellum was excluded due to incomplete coverage.

Diffusion MRI: [ ] Used [x] Not used

Preprocessing

Preprocessing software: Conn and SPM were used. Full details can be found in the manuscript.

Normalization: Normalised directly to an EPI template using SPM 12;s old norm function as this produced the best results (e.g., Calhoun et al., 2017).

Normalization template: Spm EPI template used. See above.

Noise and artifact removal: Motion parameters, CSF and White matter were removed.

Volume censoring: For the functional images, the initial 5 scans were removed. Bandpass filter was applied, specific for instantaneous phase synchrony. Movement parameters, CSF and Whitematter components were regressed out. Further information in the manuscript.

Statistical modeling & inference

Model type and settings: Multivariate, inspired by representational similarity analysis. Use of permutation and non parametric statistics.

Effect(s) tested: Similarity between behavioural and fMRI data tested (permutation), and similarity between participants (non parametric statistics.)

Specify type of analysis: ☐ Whole brain ☐ ROI-based ☐ Both

Statistic type for inference (See Eklund et al. 2016): NA; this is not an activation study. Parcellations and dynamic brain networks were used

Correction: FDR was used

Models & analysis

n/a	Involved in the study
☐	☑ Functional and/or effective connectivity
☒	☐ Graph analysis
☒	☐ Multivariate modeling or predictive analysis

Functional and/or effective connectivity: Instantenous phase syncrony.

Graph analysis: NA

Multivariate modeling and predictive analysis:

This checklist template is licensed under a Creative Commons Attribution 4.0 International License, which permits use, sharing, adaptation, distribution and reproduction in any medium or format, as long as you give appropriate credit to the original author(s) and the source, provide a link to the Creative Commons license, and indicate if changes were made. The images or other third party material in this article are included in the article's Creative Commons license, unless indicated otherwise in a credit line to the material. If material is not included in the article's Creative Commons license and your intended use is not permitted by statutory regulation or exceeds the permitted use, you will need to obtain permission directly from the copyright holder. To view a copy of this license, visit http://creativecommons.org/licenses/by/4.0/
